# Preparation and characterization of nano-galvanic bimetallic Fe/Sn nanoparticles deposited on talc and its enhanced performance in Cr(VI) removal

**DOI:** 10.1038/s41598-021-87106-0

**Published:** 2021-04-08

**Authors:** Mitra Bayat, Bahram Nasernejad, Cavus Falamaki

**Affiliations:** Department of Chemical Engineering, Amirkabir University of Technology, 15875-4413 Tehran, Iran

**Keywords:** Environmental chemistry, Nanoparticles

## Abstract

In this study, talc-supported nano-galvanic Sn doped nZVI (Talc-nZVI/Sn) bimetallic particles were successfully synthesized and utilized for Cr(VI) remediation. Talc-nZVI/Sn nanoparticles were characterized by FESEM, EDS, FTIR, XRD, zeta potential, and BET analysis. The findings verified the uniform dispersion of nZVI/Sn spherical nanoparticles on talc surface with a size of 30–200 nm, and highest specific surface area of 146.38 m^2^/g. The formation of numerous nano-galvanic cells between nZVI core and Sn shell enhanced the potential of bimetallic particles in Cr(VI) mitigation. Moreover, batch experiments were carried out to investigate optimum conditions for Cr(VI) elimination and total Cr(VI) removal was achieved in 20 min using Sn/Fe mass ratio of 6/1, the adsorbent dosage of 2 g/L, initial Cr(VI) concentration of 80 mg/L, at the acidic environment (pH = 5) and temperature of 303 K. Besides, co-existing of metallic cations turned out to facilitate the electron transfer from the nano-galvanic couple of NZVI/Sn, and suggested the revolution of bimetallic particles to trimetallic composites. The aging study of the nanocomposite confirmed its constant high activity during 60 days. The removal reaction was well described by the pseudo-second-order kinetic and the modified Langmuir isotherm models. Overall, due to the synergistic galvanic cell effect of nZVI/Sn nanoparticles and full coverage of active sites by Sn layer, Talc-nZVI/6Sn was utilized as a promising nanocomposite for fast and highly efficient Cr(VI) elimination.

## Introduction

With the rapid development of industrial activities, large amount of heavy metals have been released into the natural environment, which their continuous accumulation and high permeability have posed great threats to living organisms and environment safety^1,2^. The toxicity or carcinogenicity of heavy metal ions, such as Cr(VI), Pb(II), Cd(II), Cu(II), Zn(II), Hg(II), and Ni(II) can cause long-term damage to humans and other species^3^.

Chromium mainly exists in two oxidation states of Cr(VI) and Cr(III) among which Cr(VI) is a hundred times more toxic than the latter. Cr(VI) has been classified as priority controlled contaminant and could cause cancers and respiratory diseases in case of excessive exposure^4,5^. On the other hand, Cr(III) is significantly less toxic and even is known as an essential nutrient for humans and living organisms at trace levels. Due to low aqueous solubility and subsequent low mobility of Cr(III), the reduction of Cr(VI) to Cr(III) and successive in-situ Cr(III) adsorption or precipitation has been acknowledged as the most suitable approach for Cr(VI) mitigation^6–9^. Consequently, the employment of the improved treatment technology for fast and complete Cr(VI) reduction has drawn lots of attention^10–13^.

Technologies based on adsorption techniques have been known as adequate and straightforward approaches for Cr(VI) elimination, due to their wide adaptability and cost-effectiveness^14,15^. Various classes of adsorbents are utilized in Cr(VI) sequestration, including activated carbon^16,17^, carbon nanotube based materials^18^, graphene-based materials^19^, layered double hydroxide^20,21^, polymers and biopolymers^22–24^, Silica^25,26^ and biosorbents^27,28^. Among different adsorbent for heavy metal remediation, nanoscale zero-valent iron (nZVI) particles which have combined large specific surface area, high reactivity, abundant surface binding sites, low toxicity, and absence of secondary pollution, has been proved as an efficient and environmental-friendly materials. Accordingly, nZVI has been emerged as a promising material Cr(VI) remediation from aqueous solutions, in recent years^29–31^.

Regardless of their high efficiency in the removal of contaminants, nZVI particles have faced some limitations, including instability, strong tendency for agglomeration, and rapid oxidation. To address these issues, different modifications such as metal doping, coating the surface of nZVI particle, or immobilization of nZVI particles onto support are applied^32,33^. The wide availability, cost-effectiveness and long-term stability of clay minerals (e.g., montmorillonite, kaolinite, Bentonite, talc) have introduced them as great supporting materials for nZVI particles^6,14,32,34–36^.

Besides utilizing the preferences of clay-supported nZVI particles, the reactivity of nZVI particles has been enhanced through the deposition of a second metal catalyst (e.g., Pd, Pt, Ag, Ni, Cu) onto nZVI particles’ surface^10,37–40^. The addition of other metals would provide hydrogen catalysts or reactive electron donors, and subsequently would lead to the enhanced properties of bimetallic particles, including slower passivation of Fe^0^ surface and faster reaction kinetics^41^. Besides, bimetallic nanoparticles could form numerous nano-galvanic couples which speeded up the electron transfer of galvanic corrosion. In this regard, Fe^0^ acted as the electron donor and the deposited second metal functioned as the electron collector. Despite the enhanced performance, the wide application of bimetallic particles has been limited due to the high expense of the coated metals. Alternatively, the application of a low-cost and efficient coating metal would be an advantageous approach^37^.

Clay-supported iron based bimetallic nanoparticles, which have combined both advantages of supported and coated nanoparticles, have proved great potential in heavy metals elimination^41–43^. Despite their effectiveness in heavy metal removal, only little researches have been conducted using limited kind of clays (mainly kaolinite and montmorillonite) and metals (Ni, Pd). Talc with the chemical formula of Mg_3_Si_4_O_10_(OH)_2_ is one of the non-ionic clays with a 2:1 structure, which is composed of a sheet of magnesium hydroxide (MgO.H_2_O) located among two sheets of silicate (SiO_2_) layers. Although showing high performance as supporting material for nanoparticles, talc has been studied intensively^44,45^.

To the best of our knowledge, the application of nanocomposites containing talc as supporting surface and Sn as a coating material for Cr(VI) remediation has not been reported to date. Sn as an non-toxic, and cost-efficient metal with higher redox potential $\left( {{\text{E}}^{0} \left( {{\text{Sn}}^{2 + } /{\text{Sn}}} \right) = - 0.13\;{\text{V}}} \right)$ than Fe^0^ $\left( {{\text{E}}^{0} \left( {{\text{Fe}}^{2 + } /{\text{Fe}}} \right) = - \,0.44\;{\text{V}}} \right)$ is a superior alternative as the coating metal and the medium for enhanced electron transfer between Fe^0^ and contaminants. Herein, novel talc-supported nano-galvanic Fe/Sn bimetallic nanoparticles (Talc-nZVI/Sn) were prepared and applied for the Cr(VI) remediation from aqueous solution. A successive two-step synthesis technique was employed to achieve core–shell bimetallic nanoparticles deposited on talc. The removal of Cr(VI) was evaluated using talc, nZVI, nZVI/Sn and talc-supported nZVI/Sn with various Sn/Fe ratios. In addition, the effects of different environmental conditions such as initial Cr(VI) concentration, Talc-nZVI/Sn dosage, initial pH, and temperature on Cr(VI) removal were explored. The influence of co-existing of different metallic cation in Cr(VI) aqueous solution on the galvanic cells performance was also investigated. Moreover, the reusability and aging study of the Talc-nZVI/Sn particles were surveyed. Finally, the adsorption kinetics, adsorption isotherm, and reaction mechanism of Cr(VI) removal using Talc-nZVI/Sn were studied.

## Results and discussion

### Characterizations

**Figure 1:**
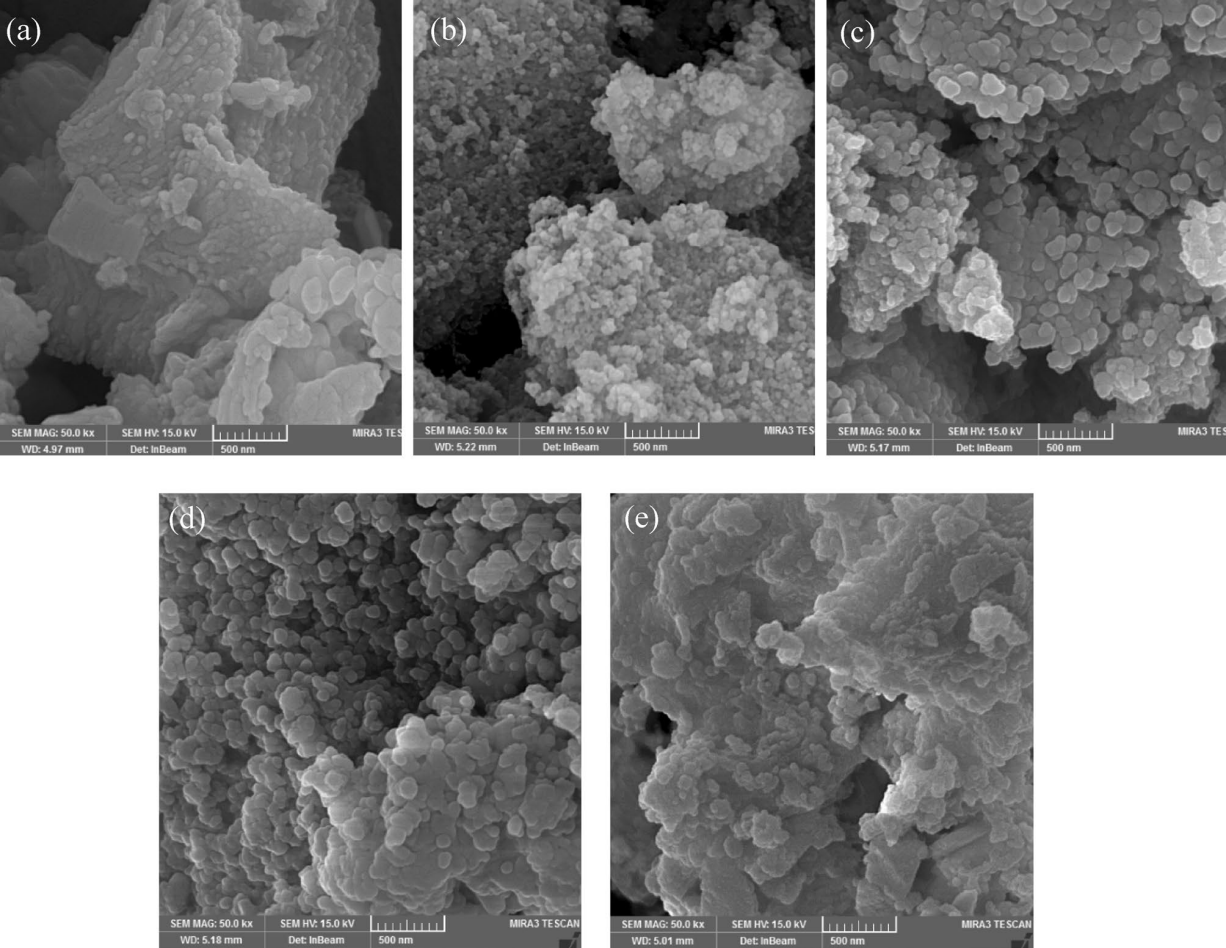
FESEM images of (**a**) talc (**b**) Talc-nZVI/1Sn (**c**) Talc-nZVI/4Sn (**d**) Talc-nZVI/6Sn (**e**) Talc-nZVI/6Sn after reaction with Cr(VI).

The morphology and surface structure of different materials, including talc, Talc-nZVI/1Sn, Talc-nZVI/4Sn, and Talc-nZVI/6Sn before and after reaction with Cr(VI) were analyzed by FESEM (Fig. 1). Talc (Fig. 1a) is primarily characterized by its smooth surface and layered structure. The FESEM images of the synthesized nanoparticles of Talc-nZVI/1Sn, Talc-nZVI/4Sn, and Talc-nZVI/6Sn, displayed approximately spherical particles, with a size of 30 to 200 nm, which was uniformly dispersed on the talc surface. Also, with an increase of the Sn/Fe mass ratio from 1 to 6, some changes in average particle size appeared. The enhancement in the Sn/Fe mass ratio, has yielded in slightly larger particles. Particles in Talc-nZVI/1Sn were mainly in the range of 30 to 100 nm, while Talc-nZVI/6Sn exhibited particles in the range of 50–200 nm. The bigger particles in Talc-nZVI/6Sn compared to Talc-nZVI/1Sn were ascribed to the increased loading of Sn, which created a thicker shell layer on nZVI cores.

**Figure 2:**
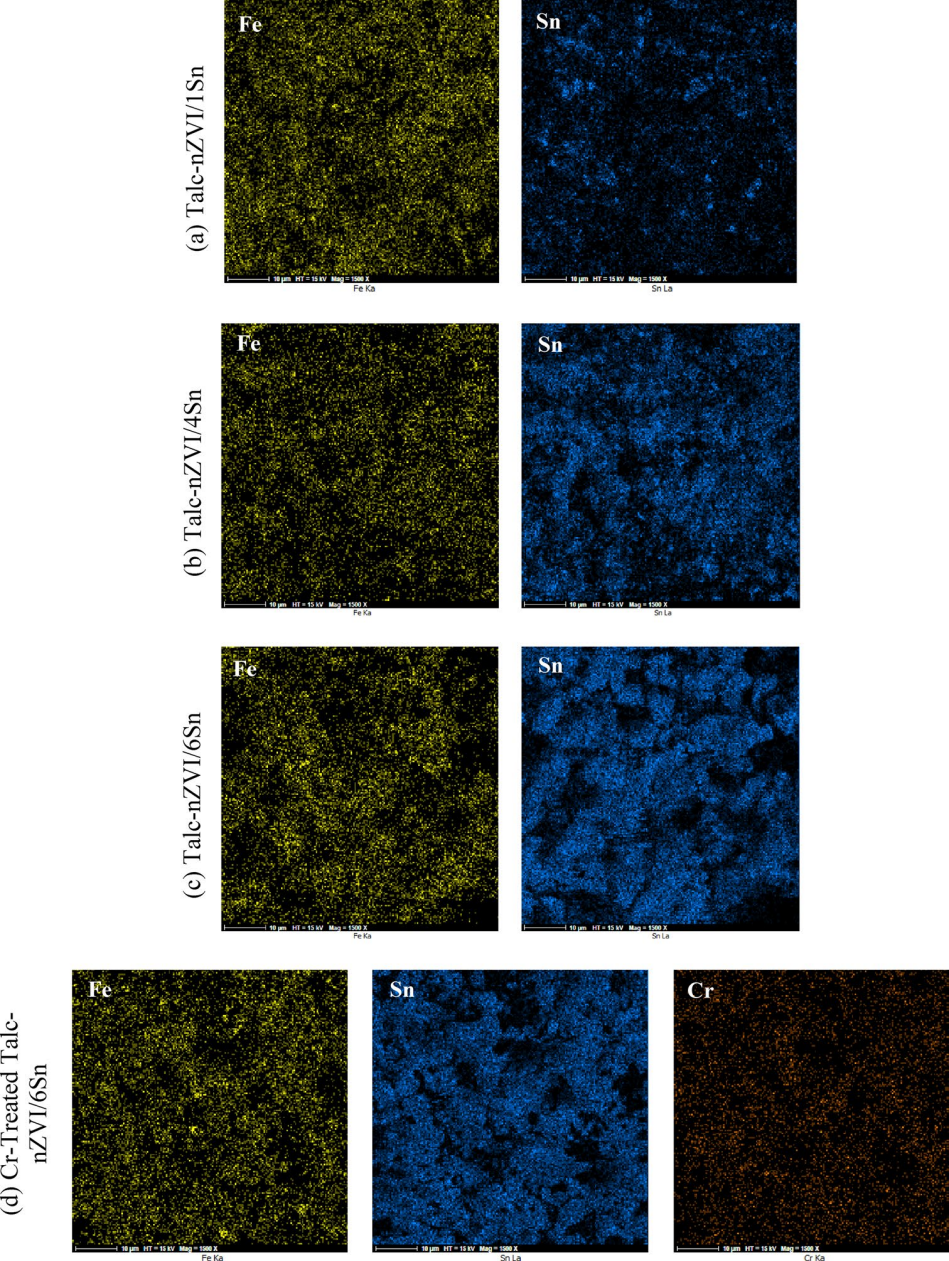
EDS mapping of Fe, Sn, and Cr in (**a**) fresh Talc-nZVI/1Sn (**b**) fresh Talc-nZVI/4Sn (**c**) fresh Talc-nZVI/6Sn and (d) Cr-treated Talc-nZVI/6Sn.

Figure 1d, e display the morphological changes of Talc-nZVI/6Sn before and after Cr(VI) removal process. The unreacted Talc-nZVI/6Sn showed particles with their explicit borders, while the reacted composite exhibited a more porous surface. This difference in surface structure could be attributed to the settlement of reaction precipitates on the outer surface of nanoparticles and within pores of talc structure. Meanwhile, the appearance of Cr-containing species is confirmed by the EDS elemental mapping of chromium on Talc-nZVI/6Sn after the removal process (Fig. 2d).

The EDS analysis was applied to clarify the elemental distribution of the different synthesized samples. EDS mapping of the adsorbents with different Sn/Fe loading of 1/1, 4/1, and 6/1 illustrated the uniform distribution of Fe and Sn over the Talc surface (Fig. 2). As can be seen, all samples showed a similar amount of Fe, whereas Sn quantity intensified significantly from 17.32 wt% in Talc-nZVI/1Sn to 64.56 wt% in Talc-nZVI/6Sn. Furthermore, the identical distribution pattern of Sn and Fe indicated the deposition of Sn shell layer on Fe fine cores^46^, becoming thicker with the increase in Sn loading from Fig. 2a–c. Also, EDS mapping of Talc-nZVI/6Sn after Cr(VI) removal process illustrated in Fig. 2d, revealed the presence and distribution of chromium on nZVI/Sn bimetallic particles.

**Figure 3:**
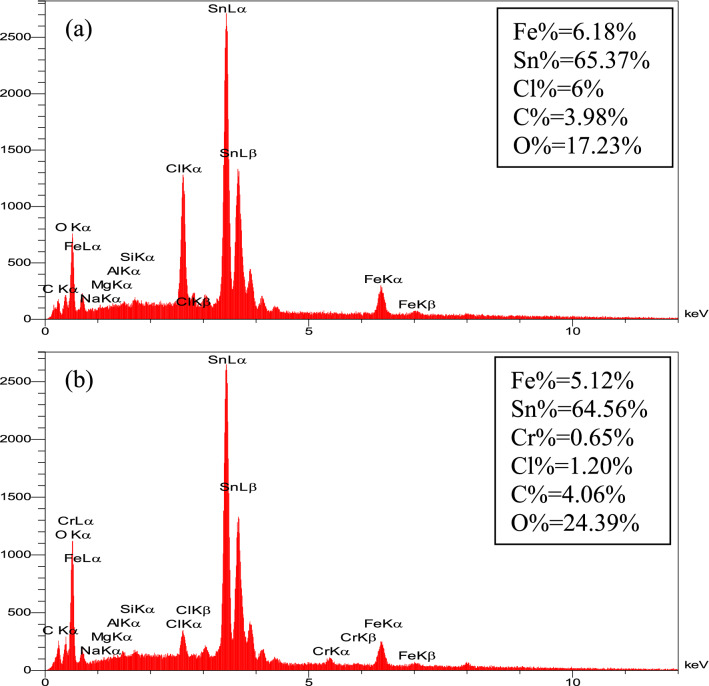
EDS spectra of (**a**) Talc-nZVI/6Sn and (**b**) Talc-nZVI/6Sn after reaction with Cr(VI).

Additionally, the elemental distribution of Talc-nZVI/6Sn before and after Cr(VI) removal was analyzed by EDS (Fig. 3). The comparison of the elemental distribution of the fresh and reacted nanocomposite pointed out the meaningful rise in oxygen content from 17.23 to 24.39% along with the appearance of Cr among the elements, in spite of the nearly constant Fe and Sn loading. The increase in O and Cr content suggested the formation and deposition of Cr(III) hydroxides precipitates on the Talc-nZVI/6Sn surface^47,48^.

The specific surface area (SSA) and total pore volume of talc and different synthesized talc-supported bimetallic particles was measured and presented in Table 1. It was revealed that the deposition of nZVI/Sn bimetallic particles on talc surface enhanced the specific surface area of the nanocomposites to such an extent that lead to a SSA of 146.38 m^2^/g (in talc-nZVI/1Sn), which was 13.5 times higher than the talc itself (10.84 m^2^/g). The highly achieved value of SSA after loading nanoparticles on talc surface was consistent with the similar clay-supported nZVI bimetallic particles^7,41,42,49^. Despite the high value of SSA and total pore volume in all the synthesized talc-supported nZVI/Sn bimetallic particles, results demonstrated that the increment of Sn loading in bimetallic nZVI/Sn particles from Sn/Fe mass ration of 1 (Talc-nZVI/1Sn) to 8 (Talc-nZVI/8Sn), would decrease the SSA and total pore volume from 146.38 m^2^/g and 0.27 cm^3^/g to 24.07 m^2^/g and 0.09 cm^3^/g, respectively. The decrease in SSA and total pore volume is ascribed to the overfilling of pores in the nanocomposite structure by the excessive Sn layer.

**Table 1:** BET characteristics of different talc-supported nZVI/Sn bimetallic particles.

Nanocomposite	Specific surface area (m^2^/g)	Total pore volume (cm^3^/g)	Pore size (nm)
Talc	10.84	0.07	27.10
Talc-nZVI/1Sn	146.38	0.27	7.38
Talc-nZVI/2Sn	117.12	0.21	7.17
Talc-nZVI/3Sn	73.59	0.18	9.78
Talc-nZVI/4Sn	32.91	0.09	10.88
Talc-nZVI/5Sn	30.67	0.1	13.04
Talc-nZVI/6Sn	26.45	0.1	15.12
Talc-nZVI/7Sn	25.86	0.1	15.40
Talc-nZVI/8Sn	24.07	0.09	15.81

**Figure 4:**
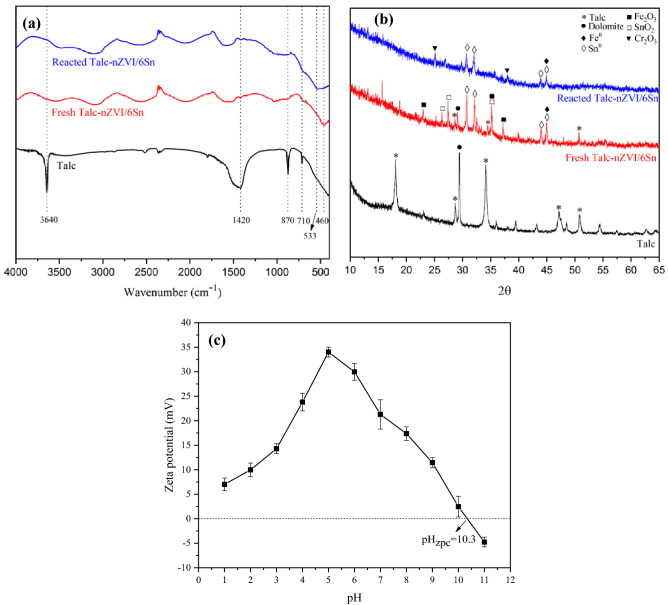
(**a**) FTIR spectra and (**b**) XRD patterns of Talc-nZVI/6Sn before and after Cr(VI) removal, (**c**) Zeta potential of Talc-nZVI/6Sn at different pH values.

The FTIR spectra of talc, Talc-nZVI/6Sn before and after reaction with hexavalent chromium are presented in Fig. 4a. Compared to FTIR spectrum of talc, the freshly synthesized Talc-nZVI/6Sn showed significant changes in peaks including weakening of the bands at 1420 and 3640 cm^-1^ corresponding the –OH group in talc. Also, the bands at 710 and 870 cm^-1^ in talc corresponding Mg–O and Si–O bands, reduced after the impregnation of Fe/Sn bimetallic particles. This was ascribed to the destruction of Mg–O and Si–O bands by alkali, produced during the synthesis of Fe/Sn bimetallic particles through the reaction between NaBH_4_ and H_2_O^50^. Furthermore, the appearance of a sharp peak at 460 cm^-1^ in the spectrum of fresh Talc-nZVI/6Sn, which was attributed to Fe–O stretches of Fe_2_O_3_ and Fe_3_O_4_, and Sn–O stretches of SnO_2_, demonstrated the impregnation of Fe/Sn bimetallic particles on talc surface and partial oxidation of particles’ surface^51,52^. Despite the similarity in the spectrum of both nanocomposites, the reacted Talc-nZVI/6Sn exhibited changes in the characteristic peaks. The enhancement in the intensity of bands corresponding to the Fe–O and Sn–O stretches at 533 cm^-1^, indicated an abundance of iron oxides and tin oxides after Cr(VI) removal. Furthermore, the band at 870 cm^−1^ was intensified due to the formation of Cr–O bond, implying the precipitation of Cr on the adsorbent surface^53^.

XRD spectra of talc, fresh and reacted Talc-nZVI/6Sn is represented in Fig. 4b. XRD pattern of talc, which has been used as supporting material mainly included major peaks at 2θ values of 18°, 28°, 34°, 47°, and 51° for talc and 29.5° for CaMg(CO_3_)_2_/dolomite^47^. The synthesized Talc-nZVI/6Sn XRD pattern primarily composed of the weakened characteristic peaks of talc and quartz and also newly formed peaks of Fe^0^ at 2θ of 44.9° and Sn^0^ at 2θ of 30°, 32°, 43.9° and 44.9°. The appearance of characteristic peaks of Fe^0^ and Sn^0^ indicated the successful formation and deposition of Fe/Sn bimetallic nanoparticles on talc surface. Furthermore, some new diffraction peaks emerged, which mainly indicated the formation of a layer of Fe_2_O_3_ (2θ = 23°, 35°, 37°) and SnO_2_ (2θ = 26°, 27°, 35°, 37°) on Fe/Sn bimetallic particles^54^. After Cr(VI) removal, peaks corresponding Fe/Sn bimetallic particles was weakened considerably, which confirmed the occurrence of redox reaction between Talc-nZVI/6Sn and Cr(VI) and precipitation of the reaction product on adsorbent surface^55^. Meanwhile, peaks at 2θ of 25° and 37.8° suggested the formation of Cr_2_O_3_ after Cr(VI) removal. Consistent with the results obtained from EDS and FTIR, these findings verified that Fe/Sn bimetallic nanoparticles has been successfully loaded on talc surface and also, chromium has been appeared on adsorbent surface after Cr(VI) removal reaction.

Also in attempt to get an insight of the adsorbent surface charge, zeta potential of the synthesized adsorbent was investigated at various pH values of 1–11 and illustrated in Fig. 4c. The results revealed that Talc-nZVI/6Sn have positive surface charge in pH range of 1–10 which is significantly beneficial towards Cr(VI) removal due to the electrostatic attraction between the nanocomposite and chromium negative oxyanions. The zero point charge (pH_zpc_) of the nanocomposite was approximately at pH of 10.3, which conformed with the result of a similar research^56^. Ji^56^ demonstrated that the deposition of a second metal on nZVI particles with higher standard redox potential that Fe, would lead to higher values of isoelectric point (pH_zpc_ > 10). Elevated zeta potential of Talc-nZVI/6Sn (+ 34 mV) at pH of 5 and high isoelectric point contributed to its stability in aqueous solution and also its great potential for elimination of negative oxyanions in a wide pH range^36,57^.

### Cr(VI) removal performance of talc-nZVI/Sn

#### Effects of Sn/Fe mass ratio

**Figure 5:**
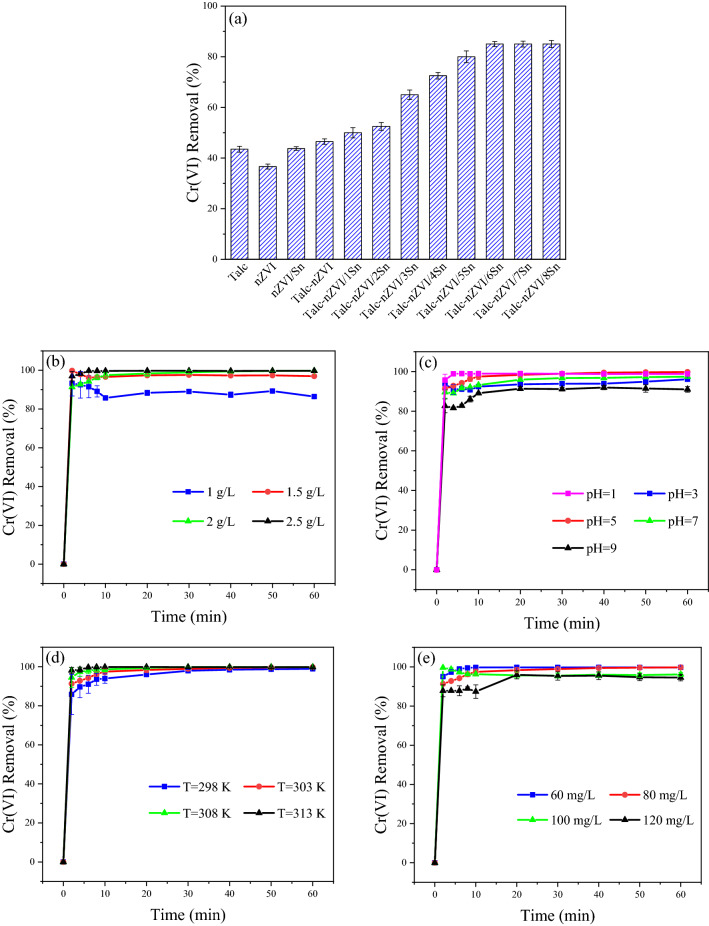
Effect of (**a**) Sn/Fe ratio (**b**) adsorbent dosage (**c**) pH (**d**) temperature and (**e**) initial Cr(VI) concentration on Cr(VI) removal.

As mentioned previously, Sn coating is used in attempt of decreasing the oxidation of nZVI particles’ surface and also facilitating the electron transfer through formation of many nano-galvanic cells between nZVI and Sn coating. So in order to investigate the extent of enhancement in Cr(VI) removal performance by the effect of Sn/Fe mass loading, a series of composites with varying Sn/Fe mass ratios were prepared, and batch experiments were carried out using 0.15 g (1 g/L) of the composites at initial chromium(VI) concentration of 80 mg/L. Furthermore, Cr(VI) elimination using talc, nZVI, nZVI/Sn, and talc-nZVI was investigated to elucidate the effectiveness of Sn loading. As shown in Fig. 5a, results revealed the weak performance of nZVI in 36.65% Cr(VI) mitigation, caused by the agglomeration of nZVI nanoparticles and surface passivation. However, immobilization of nZVI particles on talc surface and deposition of small amount of Sn coating contributed to nearly 10% and 7% enhancement in Cr(VI) removal efficiency, respectively. Additionally, results indicated that increasing Sn mass loading from Sn/Fe mass ratio of 1/1 (Talc-nZVI/Sn) to 8/1 (Talc-nZVI/8Sn) would lead to an increase in removal efficiency from 50 to 85%, respectively. Although nanocomposites with lower Sn/Fe mass ratio offered greater specific surface area (Table 1), their inefficient performance in Cr(VI) mitigation pointed out the surface passivation of the nZVI particles due to the formation of iron oxides layer. Therefore, it was clarified that addition of a second metal as coating layer played an influential part in the enhanced performance of the nanocomposite.

Talc-nZVI/Sn showed low efficiency in Cr(VI) removal. This was ascribed to the low amount of Sn coating and fast and easy oxidation of nZVI, leading to the formation of a passive layer and inhibition of electron transfer between iron nanoparticles and Cr(VI)^58^. The performance of composite enhanced gradually with increment in Sn/Fe mass ratios from 1/1 to 6/1. However, the constant Cr(VI) removal efficiency in nanocomposites with Sn/Fe mass ratios of 6/1, 7/1, and 8/1 implied that the Sn/Fe mass ratio of 6/1 had achieved the full coverage of nZVI particles, and addition of an excessive amount of Sn mass loading wouldn’t improve the performance of nanocomposite. So Talc-nZVI/6Sn, which provided the Cr(VI) removal efficiency of 85% along with the least Sn loading, was selected as the optimum adsorbent for further experiments.

#### Effects of talc-nZVI/Sn dosage

The influence of Talc-nZVI/6Sn dosage on the Cr(VI) sequestration from aqueous solution was investigated using various amount of nanocomposite (1–2.5 g/L) at a constant initial Cr(VI) concentration of 80 mg/L (Fig. 5b). It was verified that by the enhancement in Talc-nZVI/Sn dosage from 1 to 2.5 g/L, removal efficiency of Cr(VI) increased gradually from 87 to 99.7% after 60 min, while removal capacity decreased from 69.6 to 31.9 mg/g, respectively. The reason behind the improvement of removal efficiency is that applying a higher dosage of composite would provide larger surface area and more available active adsorption sites and abundant galvanic couples, which could enhance available electrons for Cr(VI) reduction^58^. However, the reduced removal capacity in higher dosage was ascribed to inter-particle aggregation, overlapping of adsorption sites, and subsequently less active surface area^15,59^.

The constant removal efficiency in the dosage of 2 and 2.5 g/L, affirmed that Talc-nZVI/6Sn dosage of 2 g/L was capable of providing sufficient active reaction sites for Cr(VI) elimination. Thus considering efficient performance on Cr(VI) removal of over 99% and low material consumption, a dosage of 2 g/L was selected as the optimum amount of adsorbent.

#### Effects of pH

The effect of initial pH as an impressive factor on adsorption characteristics was explored within the range of 1–9 at initial Cr(VI) concentration of 80 mg/L, the temperature of 303 K, and adsorbent amount of 2 g/L. As shown in Fig. 5c, despite the high Cr(VI) removal at alkaline and neutral conditions, the acidic environment was more conducive for Cr(VI) elimination. It was found that removal efficiency reached to its highest value (99.7%) at pH of 5, and reduction ability of Talc-nZVI/6Sn declined with increasing initial pH solution to 7 (97%) and 9 (91%). Similar to the great removal performance at pH of 5, the more acidic environments favored Cr(VI) sequestration and Cr(VI) removal efficiency of 99% was achieved at pH of 1. Moreover, the Cr(VI) elimination under initial pH of 2 was also investigated and Cr(VI) removal efficiency of 99% was obtained.

Since the initial solution pH strongly affects both surface charge of adsorbent and distribution of Cr(VI) species, it is among the most important influential factors on Cr(VI) removal efficiency. Solution pH impresses the process in these ways:

(i) In an aqueous solution, Cr(VI) exists in different ionic species which their relative distribution depend on total chromate concentration and the solution pH. In an acidic environment, ${\text{HCrO}}_{4}^{ - }$ is the dominant species, and in alkaline and neutral conditions, ${\text{CrO}}_{4}^{2 - }$ is the main form of hexavalent chromium^60–62^. ${\text{HCrO}}_{4}^{ - }$ is more favorable for sorption than ${\text{CrO}}_{4}^{2 - }$ due to its lower adsorption free energy and higher oxidizing capacity (Eqs. (1)-(2))^58,63^:
  1 $$ 7{\text{H}}^{ + } + {\text{HCrO}}_{4}^{ - } + 3{\text{e}}^{ - } \to 4{\text{H}}_{2} {\text{O}} + {\text{Cr}}^{3 + } \quad {\text{E}}^{0} = 1.35 \left( {\text{V}} \right) $$
  2 $$ {\text{CrO}}_{4}^{2 - } + 4{\text{H}}_{2} {\text{O}} + 3{\text{e}}^{ - } \to 5{\text{OH}}^{ - } + {\text{Cr}}\left( {{\text{OH}}} \right)_{3} \quad {\text{E}}^{0} = - 0.12 \left( {\text{V}} \right) $$
(ii) The surface charge of the adsorbent is influenced by solution pH. According to the zeta potential measurement in pH range of 1–11 (Fig. 4c), adsorbent is positively charged in both acidic and alkaline environments, in pH values lower than 10.3. The electrostatic attraction between the positively charged Talc-nZVI/6Sn and Cr(VI) oxyanions facilitated the contact between adsorbent and contaminant, and was conductive to a better removal performance. Results showed that zeta potential and electrostatic attraction reached to its greatest value at pH of 5, which provided Cr(VI) removal efficiency of 99.7%. The positive values of zeta potential and consequently electrostatic attraction between adsorbent and Cr(VI) decreased with the increasing pH, which was consistent with the obtained results in Fig. 5c.

Considering the high removal performance of nanocomposite at both pH values of 1 and 5, and the common pH values of aquatic environment in range of 5–9^2^, the pH value of 5 was selected as optimum acidic condition for further experiments. Furthermore, Table S1 represented the reduction in solution pH values after the removal process. The decrease in the final solution pH suggested that significant ${\text{OH}}^{ - }$ consumption through the precipitation process has happened.

#### Effects of temperature

To investigate the effect of temperature on Cr(VI) removal, 2 g/L of Talc-nZVI/6Sn was added to the Cr(VI) aqueous solution with various temperatures of 298, 303, 308, and 313 K. Results shown in Fig. 5d revealed that higher temperature conducted to more favorable removal performance which demonstrated that Cr(VI) removal was an endothermic process. With increasing solution temperature from 298 to 303 K, the removal rate of Cr(VI) reached from 98.5 to 99.8%, respectively. However, the further temperature rise, led to the same removal rate of Cr(VI), but in a remarkably faster process. So that at temperatures of 303 K, the removal efficiency of 99.8% is achieved after 50 min of reaction, but the time of reaching the same removal efficiency at temperatures of 308 and 313 K was 30 and 6 min, respectively. This can be ascribed to the higher mobility and diffusion rate of Cr(VI) oxyanions at elevated temperatures of 308 K and 313 K, which consequently added to the possibility of contact between chromium ions and the adsorption sites^59,64^. Therefore, results suggested that Talc-nZVI/6Sn exhibited substantial performance in removing Cr(VI) under a broad temperature range. Overall, the complete removal of Cr(VI) was obtained at the temperature of 303 K, and more temperature raised only shortened the process time. So the temperature of 303 K is selected as the optimum temperature in the following experiments.

#### Effects of initial Cr(VI) concentration

Figure 5e represents the effects of initial Cr(VI) concentration on the performance of Talc-nZVI/6Sn. The results demonstrated that increasing the initial Cr(VI) concentration from 60 to 120 mg/L, led to lower removal efficiency, whereas provided higher removal capacity. The complete Cr(VI) removal (99.8%) was achieved at initial Cr(VI) concentration of 60 and 80 mg/L, but with increasing initial Cr(VI) concentration to 100 and 120 mg/L, Cr(VI) removal declined to 96% and 95%, respectively. Obviously, a constant amount of Talc-nZVI/6Sn provided a definite amount of adsorption active sites, which would be occupied eventually by increasing Cr(VI) concentration. Furthermore, higher Cr(VI) concentration conducted to more precipitation of removal products on the adsorbent surface, hindering the electron transfer between talc-nZVI/6Sn and Cr species. Overall, both terms contributed to decreased removal efficiency in higher Cr(VI) concentrations. However, the removal capacity of 56 mg/g at Cr(VI) concentration of 120 mg/L, exhibited the great ability of Talc-nZVI/6Sn for Cr(VI) elimination^6,65^.

### Galvanic performance of Fe/Sn bimetallic nanoparticles

**Figure 6:**
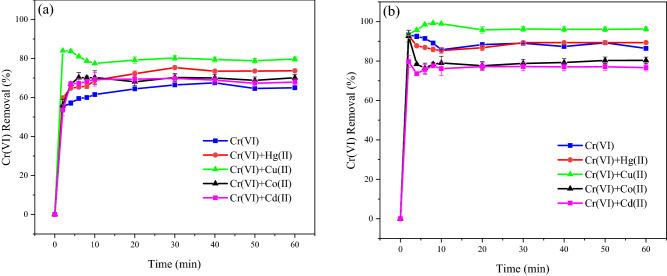
The effects of metallic cation’s presence on the Cr(VI) removal using (**a**) Talc-nZVI/3Sn and (**b**) Talc-nZVI/6Sn (Cr(VI) initial concentration: 80 mg/L, metallic cations initial concentration: 40 mg/L, nanocomposite dosage: 1 g/L).

To elucidate the galvanic performance of nZVI/Sn nanoparticles, four different metallic cations were added to the aqueous solution of Cr(VI), and their effects on Cr(VI) removal was illustrated for Talc-nZVI/3Sn (Fig. 6a) and Talc-nZVI/6Sn (Fig. 6b). Despite the expectation about the competence between the co-existing metallic cations and C(VI) anions for the limited amount of galvanic cells, results demonstrated that addition of metallic cations mainly increased the Cr(VI) removal efficiency, which confirmed their synergistic role in facilitating electron transfer to chromium oxyanions. While using Talc-nZVI/3Sn, the most enhanced Cr(VI) removal performance was achieved by the addition of metallic cations in order of Cu(II) > Hg(II) > Co(II) > Cd(II). In case of using Talc-nZVI/6Sn, the presence of Cu(II) and Hg(II) caused the Cr(VI) removal efficiency to increase from 86 to 96% and 89%, respectively. The improved performance of both nanocomposites in Cr(VI) removal could be ascribed to the adsorption and deposition of the added cationic metals to the nZVI/Sn bimetallic particles and the formation of trimetallic nZVI/Sn/M particles (M: Hg(II), Cu(II), Co(II), Cd(II)), which could facilitate and enhance the electron transfer to Cr(VI)^43^. The maximum enhanced catalytic performance of the metallic cations was related to the presence of Cu(II), which caused 14% and 10% growth in Cr(VI) removal efficiency of Talc-nZVI/3Sn and Talc-nZVI/6Sn, respectively. Additionally, the higher improvement in Cr(VI) removal capacity of Talc-nZVI/3Sn in compared to the Talc-nZVI/6Sn, could be attributed to the less coverage of nZVI particles in Talc-nZVI/3Sn which provided more nZVI cores for the deposition of metallic cations. Moreover, it was verified that the presence of Co(II) and Cd(II) lead to the decline in Cr(VI) elimination in case of using Talc-nZVI/6Sn. This decrease was related to the lower standard reduction potentials of Co(II) $\left( {{\text{E}}^{0} \left( {{\text{Co}}^{2 + } /{\text{Co}}} \right) = - 0.28{\text{ V}}} \right)$ and Cd(II) $\left( {{\text{E}}^{0} \left( {{\text{Cd}}^{2 + } /{\text{Cd}}} \right) = - 0.4{\text{ V}}} \right)$ in compared to the Sn shell layer $\left( {{\text{E}}^{0} \left( {{\text{Sn}}^{2 + } /{\text{Sn}}} \right) = - 0.13{\text{ V}}} \right)$ which ultimately would eliminate the electron flow of the nZVI/Sn nano-galvanic cells.

### Aging study and reusability of talc-nZVI/Sn

**Figure 7:**
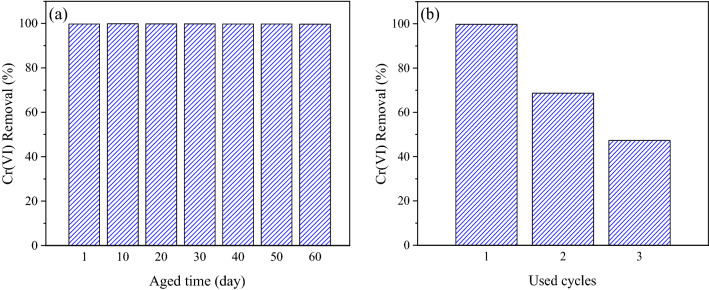
(**a**) Aging study and (**b**) reusability of Talc-nZVI/6Sn.

Regarding the protective role of Sn coating on nZVI particles, an aging study of Talc-nZVI/6Sn was carried out every 10 days to reveal the effect of time on nZVI activity (Fig. 7a). As shown in Fig. 7a, Talc-nZVI/6Sn was found to keep its high removal capacity for 2 months, which provided considerably high stability compared to previous studies^48,64^. The significant stability of Talc-nZVI/6Sn could be attributed to the full coverage of Sn coating on nZVI particles, which provided an inhibitive layer to nZVI oxidation.

Furthermore, Talc-nZVI/6Sn was repeatedly used and regenerated to investigate its reusability (Fig. 7b). Results indicated that after the first regeneration, the bimetallic nanocomposite displayed a remarkable decline in Cr(VI) removal efficiency (68%). Additionally, the second regeneration led to Cr(VI) removal efficiency of less than 50%. The low removal efficiency in Cr(VI) removal suggested the deposition of precipitates on nanocomposites, which eliminated the regeneration process.

### Adsorption kinetics

Adsorption kinetic study provides an estimation of adsorption rates along with possible reaction mechanisms^60^. Regarding the rate of solute adsorption and reactant concentration, various kinetic models can be applied to describe the rate of the adsorption process. Here, three kinetic models were used, including the pseudo-first-order (Eq. (3)), pseudo-second-order (Eq. (4)), and intraparticle diffusion (Eq. (5)) models, represented as follows^15^:

3 $$ \ln \left( {q_{e} - q_{t} } \right) = \ln \left( {q_{e} } \right) - k_{1} t $$

4 $$ t/q_{t} = 1/k_{2} q_{e}^{2} + t/q_{e} $$

5 $$ q_{t} = k_{i} t^{0.5} + C $$

where *q*_e_ and *q*_t_ (mg/g) represent the adsorption capacities at equilibrium and at time t (min), respectively; k_1_ and k_2_ are the rate constants for the pseudo-first-order and pseudo-second-order; k_i_ expresses the rate constant for the intraparticle diffusion and C is the intercept.

The Cr(VI) elimination kinetics was studied using 2 g/L talc-nZVI/6Sn at different initial Cr(VI) initial concentrations of 60, 80, 100, and 120 mg/L, and the calculated model parameters were displayed in Table 2. As result shown, the Cr(VI) removal process was in accordance with the pseudo-second-order model (R^2^ > 0.999) in compared to the other models. Also, the calculated Cr(VI) removal capacity was consistent with the experimental results. With increasing Cr(VI) concentration from 60 to 120 mg/L, the pseudo-second-order rate constant k_2_ mainly declined from 0.317 to 0.080 (g/mg min), respectively. The results revealed that chemisorption was the rate-limiting step in Cr(VI) removal process, and chemisorptive bonds, including sharing or exchanging electrons between Cr(VI) and Talc-nZVI/6Sn were involved^47,66^. The result is consistent with the FTIR and XRD findings.

**Table 2:** Kinetic parameters for Cr(VI) removal by Talc-nZVI/6Sn.

Kinetic model	Initial Cr(VI) concentration (mg/L)	Parameters		
Pseudo-first order		q_e_ (mg/g)	k_1_ (1/min)	R^2^
	60	29.826	1.545	0.9995
	80	39.024	1.324	0.9940
	100	48.123	3.955	0.9976
	120	55.261	1.485	0.9849
Pseudo-second order		q_e_ (mg/g)	k_2_ (g/mg min)	R^2^
	60	30.078	0.317	0.9998
	80	39.760	0.117	0.9988
	100	48.565	0.519	0.9999
	120	56.470	0.080	0.9905
Intraparticle diffusion		C	k_i_ (mg/(g min^0.5^))	R^2^
	60	19.336	1.959	0.2199
	80	24.336	2.784	0.2789
	100	24.161	4.387	0.3385
	120	33.998	4.090	0.3035

### Adsorption isotherm analysis

In an attempt to better understand the adsorption of Cr(VI) by Talc-nZVI/6Sn, four common isotherm models were applied to fit the experimental data, and the results were summarized in Table 3. Due to some shortcomings of the Langmuir isotherm model for describing the interaction mechanisms of the pollutant and adsorbent in the liquid phase, the modified Langmuir model was employed^67^. So, adsorption isotherm models including modified Langmuir (Eq. (6)), Freundlich (Eq. (7)), Sips (Eq. (8)), and Temkin (Eq. (9)) were used, as following^68,69^:

6 $$ q_{e} = \frac{{q_{m} K_{ML} C_{e} }}{{\left( {C_{s} - C_{e} } \right) + K_{ML} C_{e} }} $$

7 $$ q_{e} = K_{F} C_{e}^{{1/n_{F} }} $$

8 $$ q_{e} = \frac{{q_{m} (K_{S} C_{e} )^{{n_{S} }} }}{{1 + (K_{S} C_{e} )^{{n_{S} }} }} $$

9 $$ q_{e} = \frac{RT}{b}\ln K_{T} + \frac{RT}{b}\ln C_{e} $$

**Table 3:** Adsorption isotherm model parameters for Cr(VI) removal by Talc-nZVI/Sn.

Isotherm model	Parameters			R^2^
Modified Langmuir	q_m_ (mg/g)	K_ML_ × 10^6^		
	53.52428	0.708157		0.97958
Freundlich	K_F_ (mg^1−1/n^ L^1/n^/g)	1/n		
	43.67506	0.121405		0.97581
Sips	q_m_ (mg/g)	K_S_ (L/mg)	n_s_	
	43.4401	24.95368	1.05354	0.97309
Temkin	K_T_ (L/g)	b (J/mol)		
	5036.517	480.5157		0.97692

where q_e_ is the adsorption capacity at equilibrium (mg/g), q_m_ is the maximum adsorption capacity, and C_e_ represents the equilibrium concentration of Cr(VI) (mg/L). K_ML_ and K_F_ (mg^1−1/nF^ L^1/nF^/g) are the modified Langmuir and Freundlich constants, respectively, and $1/n_{F}$ is the heterogeneity factor. Also, in modified Langmuir model C_s_ represents saturated concentration of solutes. In Sips model, which is a combination of Langmuir and Freundlich isotherm models, K_S_ expresses the Sips constant, and $n_{s}$ is the Sips isotherm exponent. Also, in the Temkin equation, b is the Temkin constant related to the heat of adsorption (J/mol); R represents the universal gas constant (J/mol K); T is the temperature (K); and K_T_ expresses the Temkin isotherm constant (L/g).

Results verified that the modified Langmuir isotherm model yielded the best fitting results (R^2^ = 0.97958) in compared to the other models (Table 3). The results are in agreement with previous studies on Cr(VI) removal using similar adsorbents. Similar to the conventional Langmuir model, the modified Langmuir isotherm model indicates that adsorption of Cr(V) on the Talc-nZVI/6Sn occurred through monolayer adsorption, and all active sites were homogeneous and energetically equivalent. However, the modified Langmuir model takes the role of adsorbate concentration into consideration, and emphasizes that the desorption rate is highly affected by the bulk adsorbate concentration^69^. Furthermore, the modified Langmuir isotherm model assumes that each site can bind a single adsorbate molecule, and there is no interaction between two adsorbed pollutant molecules. Also, the modified Langmuir model implies that adsorption processes are controlled by chemical reactions^29,70^.

### Cr(VI) removal mechanism

**Figure 8:**
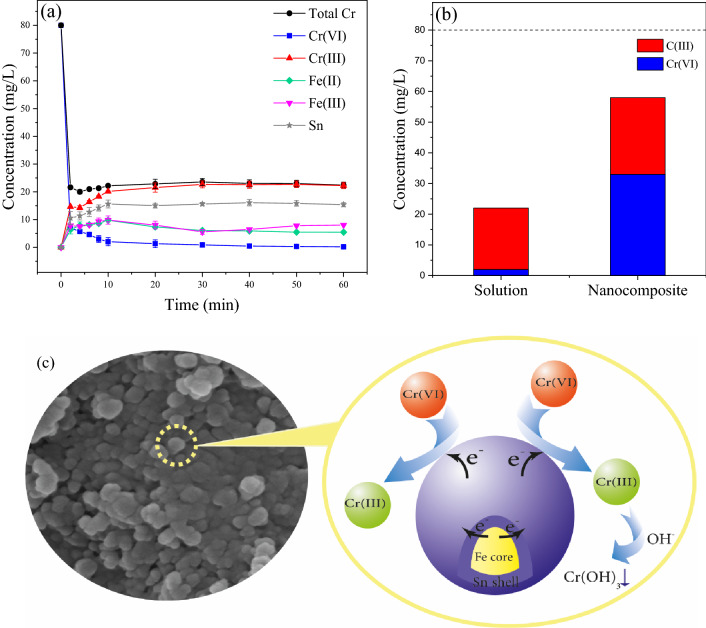
(**a**) The variation of Cr, Fe, and Sn concentrations in aqueous solution (initial Cr(VI) concentration: 80 mg/L, temperature: 30 °C, pH: 5, adsorbent dosage: 2 g/L) (**b**) valence states of Cr in aqueous solution and nanocomposite after Cr(VI) removal process (**c**) Proposed mechanism of Cr(VI) removal by Talc-nZVI/6Sn.

The variations in Cr, Fe, and Sn concentrations and valence states in aqueous solution during reaction time using Talc-nZVI/6Sn was illustrated in Fig. 8a. Total Cr concentration showed a sharp decrease after 4 min of the reaction, and reached to the constant value of 22 mg/L, suggesting that 58 mg/L of chromium was combined with Talc-nZVI/6Sn in forms of Cr(VI) and/or Cr(III). Likewise, hexavalent chromium followed a similar trend to total chromium, but reached to a much less value of 0.1 mg/L after 60 min. Meanwhile, Cr(III) increased gradually and became the dominant form of chromium in the solution. In addition, the presence of Fe (13 mg/L) and Sn (15 mg/L) in the solution indicated that Fe and Sn was oxidized and released to the solution. According to Fig. 8b on the valence distribution of chromium in aqueous solution and nanocomposite, it is revealed that following the adsorption of Cr(VI) on the nanocomposite surface, approximately 60% of Cr(VI) was reduced to Cr(III) and the other fraction remained in their adsorbed form. Meanwhile, the produced Cr(III) existed in forms of both precipitations on the adsorbent and dissolved in aqueous solution.

Thus, considering the above discussions and the findings from XRD, FTIR, and SEM–EDS analysis of the nanocomposite before and after reaction with pollutant, it was concluded that Cr(VI) removal by Talc-nZVI/6Sn involved multiple processes of adsorption-reduction and precipitation, reinforced by the numerous nano-galvanic cell effects of nZVI/Sn bimetallic particles (Fig. 8c). The reduction of contaminant occurred through two approaches. First, numerous nano-galvanic Fe/Sn cells was formed, wherein Fe $\left( {{\text{E}}^{0} \left( {{\text{Fe}}^{2 + } /{\text{Fe}}} \right) = - 0.44{\text{ V}}} \right)$ and Sn $\left( {{\text{E}}^{0} \left( {{\text{Sn}}^{2 + } /{\text{Sn}}} \right) = - 0.13{\text{ V}}} \right)$ acted as anode and cathode, respectively. The galvanic cell effects catalytically enhanced the electron transfer from Fe to Sn and ultimately to Cr(VI) ions. Simultaneously, the transferred electron reduced Cr(VI) to Cr(III)^71^. The possible reactions dominated by galvanic interactions are suggested by Eqs. (10)-(11) as follows:

10 $$ 3{\text{Fe}}^{0} - {\text{Sn}}^{0} + 2{\text{HCrO}}_{4}^{ - } + 14{\text{H}}^{ + } \to 3{\text{Fe}}^{0} - {\text{Sn}}^{2 + } + 2{\text{Cr}}^{3 + } + 8{\text{H}}_{2} {\text{O}} $$

11 $$ 3{\text{Fe}}^{0} - {\text{Sn}}^{0} + 2{\text{CrO}}_{4}^{2 - } + 16{\text{H}}^{ + } \to 3{\text{Fe}}^{0} - {\text{Sn}}^{2 + } + 2{\text{Cr}}^{3 + } + 8{\text{H}}_{2} {\text{O}} $$

Secondly, the presence of dissolved Fe(II) and Fe(III) suggested that nZVI particles directly participated in Cr(VI) reduction (Eqs. (12)–(13)), leading to the diffusion of Fe species into the solution. Also, based on the above results, nearly half of the produced Cr(III) remained as precipitations on the nanocomposite surface and the other fraction was released to aqueous solution ultimately (Eq. (14)).

12 $$ 3{\text{Fe}}^{0} + 2{\text{HCrO}}_{4}^{ - } + 14{\text{H}}^{ + } \to 3{\text{Fe}}^{2 + } + 2{\text{Cr}}^{3 + } + 8{\text{H}}_{2} {\text{O}} $$

13 $$ 3{\text{Fe}}^{2 + } + {\text{HCrO}}_{4}^{ - } + 7{\text{H}}^{ + } \to 3{\text{Fe}}^{3 + } + {\text{Cr}}^{3 + } + 4{\text{H}}_{2} {\text{O}} $$

14 $$ 2{\text{Cr}}^{3 + } + 6{\text{OH}}^{ - } \leftrightarrow 2{\text{Cr}}\left( {{\text{OH}}} \right)_{3} \left( {\text{s}} \right) \leftrightarrow {\text{Cr}}_{2} {\text{O}}_{3} \left( {\text{s}} \right) + 3{\text{H}}_{2} {\text{O}} $$

### Comparison of Cr(VI) removal by similar nZVI-based materials

A comparison between the removal capacity of Talc-nZVI/6Sn and some similar previous researches using supported-nZVI particles is presented at Table 4. It could be found that Talc-nZVI/6Sn provided higher and in some cases similar Cr(VI) removal capacity. The high removal capacity (56 mg/g), great longevity, simplicity of synthesis method, and affordable raw materials used as support and coating of nZVI particles, demonstrated the high capability of Talc-nZVI/6Sn in remediation of Cr-contaminated resources.

**Table 4:** Comparison of maximum removal capacity of Cr(VI) by supported-nZVI materials.

Materials	Material dosage (g/L)	pH	Removal capacity (mg/g)	Source
Bentonite-supported nZVI	10	6	7.3	^72^
Sepiolite-supported nZVI	1.6	6	43.86	^6^
Amino-functionalized vermiculite-supported nZVI	0.625	5	59.17	^36^
Montmorillonite-supported nZVI	0.22	5.5	31.65	^73^
Kaolin-supported nZVI	2	4	33.39	^74^
Bentonite-supported organosolv lignin-stabilized nZVI	1	3	50	^75^
Graphene nanosheets-supported nZVI	1	7	21.72	^5^
Biochar-supported nZVI	1.5	4	35.30	^76^
Biochar-supported nZVI	–	4	40	^77^
Talc-supported nZVI/Sn	2	5	56	This study

## Conclusions

Talc supported nano-galvanic nZVI/Sn bimetallic particles were prepared and utilized for hexavalent chromium elimination. Nanocomposites with various Sn/Fe mass ratios from 1/1 to 8/1 were synthesized and optimized for Cr(VI) removal reaction. FESEM analysis, EDS mapping, and EDS spectra of the nanomaterials indicated that the core–shell bimetallic nZVI/Sn particles were deposited on talc layered structure while the shell thickness increased with increment in Sn/Fe mass ratio. Furthermore, the effects of other influential factors, including nanocomposite dosage, pH, temperature, and initial Cr(VI) concentration on the process efficiency were investigated. The highest Cr(VI) removal efficiency of 99.8% was achieved using Sn/Fe mass ratio of 6/1, the adsorbent dosage of 2 g/L, pH of 5, the temperature of 303 K, and initial Cr(VI) concentration of 80 mg/L, after 10 min of reaction. Monitoring the impacts of co-existing metallic cations on the galvanic performance of Talc-nZVI/3Sn and Talc-nZVI/6Sn revealed the synergistic effects of cations through formation of trimetallic particles which led to 14% and 10% increase in Cr(VI) removal efficiency, respectively. Talc-nZVI/6Sn preserved the high capacity for Cr(VI) elimination for 60 days due to the thick and protective layer of Sn on nZVI active sites. However, the ability of nanocomposite for regeneration was limited, and its efficiency declined to less than 50% after 2 times of regeneration. The reaction results were in accordance with the pseudo-second-order kinetic model and the modified Langmuir isotherm model in which Cr(VI) removal was controlled by chemical reactions. Finally, the Cr(VI) removal process was speculated to follow adsorption, reduction, and precipitation of products, which was enhanced by the galvanic cell effects of nZVI/Sn bimetallic particles.

## Materials and methods

### Chemicals

A commercial Iranian talc with a mesh size of 325 was used. All chemicals including ferric chloride hexahydrate $\left( {{\text{FeCl}}_{3} .6{\text{H}}_{2} {\text{O}}} \right)$, tin(II) Chloride dihydrate $\left( {{\text{SnCl}}_{2} .2{\text{H}}_{2} {\text{O}}} \right)$, potassium dichromate $\left( {{\text{K}}_{2} {\text{Cr}}_{2} {\text{O}}_{7} } \right)$, sodium borohydride $\left( {{\text{NaBH}}_{4} } \right)$, diphenylcarbazide $\left( {{\text{C}}_{13} {\text{H}}_{14} {\text{N}}_{4} {\text{O}}} \right)$, 1,10-phenanthroline $\left( {{\text{C}}_{12} {\text{H}}_{8} {\text{N}}_{2} } \right)$, ethanol (99.7%), sodium hydroxide $\left( {{\text{NaOH}}} \right)$, hydrochloric acid $\left( {{\text{HCl}}} \right)$, sulfuric acid $\left( {{\text{H}}_{2} {\text{SO}}_{4} } \right)$ and metallic cations (${\text{HgCl}}_{2}$, ${\text{CuCl}}_{2} .2{\text{H}}_{2} {\text{O}}$, ${\text{CoCl}}_{2} .6{\text{H}}_{2} {\text{O}}$, ${\text{CdCl}}_{2} .{\text{H}}_{2} {\text{O}}$) were of analytical grade and were purchased from Merck. All the chemicals were used as received from the supplier without further purification.

### Preparation of nZVI and talc-nZVI/Sn nanoparticles

Chemical reduction, as the most frequent synthesis technique of nZVI has been utilized to prepare the bimetallic particles, due to its simplicity and homogeneous structure of the products^33^. Based on their synthesis method, bimetallic nanoparticles would have different structures such as alloy, core–shell, and contact aggregate^39^. Since this study aimed to achieve a core–shell structure deposited on talc, a successive two-step reduction method was employed. In this approach, Fe^0^ cores are formed primarily on the talc surface and second metal (Sn) with higher redox potential is added subsequently. The second metal reduction happened through the redox process between Fe^0^ and Sn^2+^, and the precipitation of the Sn on the Fe^0^ cores ultimately formed Fe/Sn core–shell bimetallic structures^71^.

At first, talc was washed, filtered, and dried at 90 °C overnight prior to the experiment. ${\text{FeCl}}_{3} .6{\text{H}}_{2} {\text{O}}$ and talc (mass ratio of talc:Fe^3+^ at 2:1) were dissolved in 100 mL solution of ethanol and deionized water (70%, v/v) and mixed for 30 min and 200 rpm in a three-neck flask. Then, the freshly prepared reducing agent (NaBH_4_, 50 mL, 1.07 M) was added dropwise (1–2 drops in every second) into the solution along with vigorous stirring. Immediately with the addition of the first drop of the sodium borohydride, the color of the solution turned black. After addition of all of NaBH_4_ solution, mixing continued for another 30 min to ensure the maximum yield in nZVI production and deposition on talc. Theoretically, the reaction in the process occurred in three following steps [Eqs. (15) to (17)]^34^:

15 $$ 4{\text{Fe}}^{3 + } + {\text{BH}}_{4}^{ - } + 3{\text{H}}_{2} {\text{O}} \to 4{\text{Fe}}^{2 + } + {\text{H}}_{2} {\text{BO}}_{3}^{ - } + 4{\text{H}}^{ + } + 2{\text{H}}_{2} \uparrow $$

16 $$ 2{\text{Fe}}^{2 + } + {\text{BH}}_{4}^{ - } + 3{\text{H}}_{2} {\text{O}} \to 2{\text{Fe}}^{0} \downarrow + {\text{H}}_{2} {\text{BO}}_{3}^{ - } + 4{\text{H}}^{ + } + 2{\text{H}}_{2} \uparrow $$

17 $$ 4{\text{Fe}}^{3 + } + 3{\text{BH}}_{4}^{ - } + 9{\text{H}}_{2} {\text{O}} \to 4{\text{Fe}}^{0} \downarrow + 3{\text{H}}_{2} {\text{BO}}_{3}^{ - } + 12{\text{H}}^{ + } + 6{\text{H}}_{2} \uparrow $$

Afterward, ${\text{SnCl}}_{2} .2{\text{H}}_{2} {\text{O}}$ was added to the mixture with different Sn/Fe mass ratios (1/1, 2/1, 3/1, 4/1, 6/1, 7/1, 8/1) and stayed under continuous stirring for 30 min. Finally, the prepared nanoparticles were separated using vacuum filtration, washed three times with ethanol, and dried for 12 h at 60 °C. The whole process was performed under continuous nitrogen gas purging.

### Batch experiments

The batch experiments were conducted in duplicates in a glass beaker containing 150 mL of Cr(VI) solution. Basically, the synthesized nanocomposite were added to the beaker that contained Cr(VI) solution at 303 K and pH of 5, under continuous stirring (300 rpm) for 60 min. At specific time intervals, the supernatant was taken out and immediately filtered to separate the particles through a 0.45 mm filter membrane. Afterward, dilution and acidification of the sample were carried out to measure the residual concentrations of Cr(VI). The effects of Sn/Fe mass ratio (1/1, 2/1, 3/1, 4/1, 6/1, 7/1, 8/1), Talc-nZVI/Sn dosage (1–2.5 g/L), pH (1–9), temperature (298–313 K) and initial Cr(VI) concentration (60–120 mg/L) on the removal performance were studied.

A stock solution of Cr(VI) (1000 mg/L) was prepared and diluted with deionized water to achieve the desired concentration of Cr(VI) solution. The initial pH values of the Cr(VI) solution was adjusted using 0.1 M HCl or 0.1 M NaOH.

The removal efficiency and the removal capacity of Cr(VI) at equilibrium were determined by the following Eqs. (18)–(19):

18 $$ R = \frac{{C_{0} - C_{t} }}{{C_{0} }} \times 100\% $$

19 $$ q_{t} = \frac{{\left( {C_{0} - C_{t} } \right) \times V}}{m} $$

where R is the removal efficiency of Cr(VI), and C_0_ and C_t_ (mg/L) are the initial and equilibrium concentrations of Cr(VI), respectively. Also, q_t_ (mg/g) represents the removal capacity of hexavalent chromium at a contact time of t (min). Finally, V (L) is the volume of Cr(VI) solution tested, and m (g) represents the weight of Talc-nZVI/Sn added to the solution.

In attempt to clarify the valence distribution of chromium between aqueous solution and adsorbent, the reacted nanocomposite was washed 3 times with deionized water and then was added to 1 M HCl solution and stirred. The concentration of the chromium species was determined in the samples, after filtration^58^.

In addition, the effects of co-existing metallic cations with different redox potentials on the performance of nanocomposite in Cr(VI) elimination were investigated. In these experiments, the presence of metallic cations including Hg(II), Cu(II), Co(II) and Cd(II) with initial concentration of 40 mg/L and Cr(VI) with initial concentration of 80 mg/L were evaluated using nanocomposite amount of 1 g/L at pH of 5 and T of 303 K.

Also, the reusability of the synthesized adsorbent was investigated through sequential Cr(VI) remediation and adsorbent regeneration. In this regard, after Cr(VI) removal reaction, Talc-nZVI/Sn was collected and chemically reduced using NaBH_4_ solution (50 mL, 1.07 M) under continuous stirring for 60 min. Afterwards, the regenerated adsorbent was washed with ethanol for three times, dried for 12 h at 60 °C, and used for Cr(VI) remediation. Finally, the effect of aging on the activity of the synthesized Talc-nZVI/Sn was investigated for 60 days (every 10 days). All of the experiments were conducted in duplicate.

### Characterization and analysis methods

The surface morphology and elemental distribution of the different synthesized nanocomposites were obtained using field emission scanning electron microscopy (FESEM, MIRA3 TESCAN, USA) equipped with energy dispersive spectroscopy (FESEM-EDS) system. The crystal structure of the fresh and reacted nanocomposites were analyzed by X-ray diffraction (XRD, X-Ray Explorer, GNR, Italy) using Cu-Kα radiation. An infrared spectrometer was used to obtain the Fourier transform infrared spectroscopy (FTIR) spectra of Talc-nZVI/6Sn before and after Cr(VI) removal reaction (Nicolet iS10, Thermo Scientific, USA). Zeta potentials were measured at various pH values by Wallis zeta potential analyzer (Cordouan Technologies, France) at 25 °C.

Brunauer–Emmett–Teller (BET) N_2_ adsorption analysis was performed to determine the specific surface area, total pore volume, and pore size of the particles using a surface area analyzer (Autosorb-1-MP, Quantachrome Instruments, USA). The Cr(VI) concentration was detected by the 1,5-diphenylcarbazide method with a UV–vis spectrophotometer at a wavelength of 540 nm. Also, the concentration of Fe(II) was determined through the 1,10-phenanthroline method using UV–vis spectrophotometry at wavelength of 510 nm. Total Cr, total Fe, and Sn concentrations were determined using flame atomic absorbance spectrometer. The Cr(III) and Fe(III) concentrations were calculated by subtracting Cr(VI) from total Cr concentration, and Fe(II) from total Fe concentration, respectively.

## Supplementary Information

Supplementary Information 1.
