# Supplementary material for: Preparation and characterization of nano-galvanic bimetallic Fe/Sn nanoparticles deposited on talc and its enhanced performance in Cr(VI) removal
Source: Sci Rep. 2021 Apr 8;11:7715. doi: 10.1038/s41598-021-87106-0 (PMC8032741; doi:10.1038/s41598-021-87106-0)
Supplement: Supplementary file 1 — Supplementary Information 1. [file 41598_2021_87106_MOESM1_ESM.docx]

# Supplementary Information

#### **Table S1.** solution pH before and after Cr(VI) removal

| Initial pH | 1 | 3 | 5 | 7 | 9 |
| --- | --- | --- | --- | --- | --- |
| Final pH | 1.08 | 2.97 | 3.02 | 3.26 | 3.37 |
